# Maternal and offspring genome-wide association study of C-reactive protein reveals limited polygenic association with gestational diabetes mellitus

**DOI:** 10.1186/s12864-026-12878-6

**Published:** 2026-09-24

**Authors:** Yu Zhang, Amy Moore, Kelli K. Ryckman, Qi Yan, Rafael F. Guerrero, Ming Li, Robert M. Silver, Juhua Luo, Lynn M Yee, Uma M. Reddy, Maisa N. Feghali, Judith Chung, David M. Haas, Kok Lim Kua, Nianjun Liu

**Affiliations:** 1 Department of Epidemiology and Biostatistics, Indiana University School of Public Health-Bloomington, Bloomington, IN United States of America; 2 Division of Biostatistics and Epidemiology, RTI International, Atlanta, GA United States of America; 3 Department of Obstetrics and Gynecology, Columbia University, New York, NY United States of America; 4 Department of Biological Sciences, North Carolina State University, Raleigh, NC United States of America; 5 Division of Maternal-Fetal Medicine, Department of Obstetrics and Gynecology, University of Utah Health Sciences Center, Salt Lake City, UT United States of America; 6 Division of Maternal-Fetal Medicine, Department of Obstetrics and Gynecology, Northwestern University Feinberg School of Medicine, Chicago, IL United States of America; 7 Department of Obstetrics, Gynecology and Reproductive Sciences, University of Pittsburgh School of Medicine, Pittsburgh, PA United States of America; 8 Division of Maternal-Fetal Medicine, Department of Obstetrics and Gynecology, School of Medicine, University of California, Irvine, CA United States of America; 9 Department of Obstetrics and Gynecology, Indiana University School of Medicine, Indianapolis, IN United States of America; 10 Department of Pediatrics, Division of Neonatal-Perinatal Medicine, Indiana University School of Medicine, Indianapolis, IN United States of America

**Keywords:** Early pregnancy, Maternal, Offspring, CRP, GWAS, PRS, GDM

## Abstract

**Background:**

C-reactive protein (CRP) is a well-established biomarker of systemic inflammation. In pregnancy, several studies show association of elevated CRP with gestational diabetes mellitus (GDM). However, the genetic contributions of CRP levels during early pregnancy and their potential association with GDM remain largely understudied. The Nulliparous Pregnancy Outcomes Study: Monitoring Mothers-to-Be (nuMoM2b) provides a unique opportunity to investigate these questions due to its rich design, including early pregnancy biospecimens, genome-wide genotype data, and detailed clinical outcomes across multiple ancestry groups.

**Results:**

We performed genome-wide association studies (GWAS) of first-trimester CRP levels using maternal (*n* = 4,326) and offspring (*n* = 2,140) genotypes from the nuMoM2b cohort. In the European-ancestry maternal sub-cohort of nuMoM2b, three genome-wide significant loci (*CRP*, *LEPR*, and *HNF1A*) were associated with early pregnancy CRP levels, consistent with findings from previous GWAS in non-pregnant populations. Analysis of the full multi-ancestry maternal cohort identified two additional loci (*ENSG00000257703* and *APOC1*) associated with CRP levels. No genome-wide significant associations were detected in the offspring GWAS of CRP levels. Linkage disequilibrium score regression did not detect a significant genetic correlation between CRP and GDM using the nuMoM2b CRP GWAS. In contrast, a significant correlation was observed when using an external large population-based CRP GWAS, suggesting that genetic overlap between CRP and GDM may vary across study contexts. However, Polygenic risk scores for CRP derived from either GWAS source were not significantly associated with GDM risk in the nuMoM2b cohort.

**Conclusions:**

These findings provide insights into the genetic architecture of CRP levels during early pregnancy and illustrate the potential value of pregnancy-specific genomic studies for improving understanding of inflammatory processes relevant to maternal-fetal health. They also highlight the need for larger pregnancy cohorts to better understand the relationship between inflammation and metabolic complications during pregnancy.

**Supplementary Information:**

The online version contains supplementary material available at https://doi.org/10.1186/s12864-026-12878-6.

## Background

C-reactive protein (CRP) is a widely recognized biomarker of systemic inflammation and has been extensively studied for its ability to predict cardiovascular diseases, metabolic disorders, and adverse pregnancy outcomes [1–5]. During pregnancy, maternal immune and inflammatory processes are tightly regulated to support fetal development [6, 7]. Disruption of this balance, especially during early pregnancy, can elevate CRP levels, which have been associated with an increased risk of gestational diabetes mellitus (GDM) [8, 9], preeclampsia [10], and preterm birth [11–13]. These conditions pose significant long-term health risks to both mother and child, including higher likelihood of cardiovascular and metabolic diseases [11, 14]. Understanding how genetic variants influence CRP levels during pregnancy may offer potential avenues for clinical intervention and improving maternal-fetal health.

Several genome-wide association studies (GWAS) have identified genetic variants associated with CRP levels in general populations [15–18]. However, the genetic variants influencing CRP levels during early pregnancy remain largely understudied. This gap is critical, as early pregnancy represents a unique physiological and immunological state that is different from non-pregnant individuals and therefore may require unique genetic regulation of inflammation and CRP production. Prior research showed strong phenotypic associations between higher CRP levels during early pregnancy and increased risk of GDM [9]. However, it remains unclear whether genetic variants that influence CRP levels also contribute to GDM risk.

In addition to maternal genetic influences, fetal genetic variations may also contribute to maternal physiological traits during pregnancy. This phenomenon, sometimes referred to as “fetal drive,” hypothesizes that genetic variants inherited by the fetus from the mother can influence maternal outcomes such as blood pressure and metabolic regulation [19–22]. Prior studies have demonstrated associations between fetal genetic variations and maternal blood pressure after adjusting for maternal genotype, supporting the idea that fetal genes can exert systemic effects on the maternal environment. Whether fetal genetic variation similarly affects maternal CRP during early pregnancy, however, has not yet been investigated.

To address these gaps, we investigated the genetic contributions to CRP levels measured during early pregnancy using maternal genome-wide genotype data from the Nulliparous Pregnancy Outcomes Study: Monitoring Mothers-to-Be (nuMoM2b) cohort [23]—a large, multi-center, prospective study of over 10,000 nulliparous women in the United States with comprehensive clinical, biomarker, and genomic data. Previous research within the nuMoM2b cohort demonstrated a strong phenotypic association between elevated early pregnancy CRP levels and subsequent risk of GDM [11, 24]. To evaluate the potential genetic link between CRP and GDM, we performed linkage disequilibrium score regression (LDSC) to assess shared genome-wide genetic architecture and constructed polygenic risk scores (PRS) for CRP to test whether maternal genetic predisposition to higher CRP levels was associated with increased risk of GDM. In addition, we conducted Mendelian randomization (MR) analyses to evaluate whether genetically predicted CRP levels have a causal effect on GDM risk. Finally, given the potential influence of offspring genetic variation on maternal physiology [22, 24], we conducted an additional GWAS of maternal CRP levels using offspring cord blood-derived genotypes, to explore whether offspring genetic variations contribute to maternal inflammation during early pregnancy.

## Materials and methods

### Study population

The study was conducted within the nuMoM2b cohort, a large, multicenter, prospective study that enrolled 10,038 nulliparous pregnant women between 2010 and 2013 at eight clinical sites across the United States [23]. Participants were enrolled during the first trimester of pregnancy (gestational age 6 to 13 + 6 weeks), and comprehensive data collection included demographic information, clinical assessments, and biospecimen sampling. A total of 4,502 maternal serum samples was collected during this visit and processed under standardized protocols in a centralized biorepository to ensure sample integrity. Serum CRP concentrations during the first trimester (mg/L) were measured using the Beckman Coulter AU System High-Sensitivity C-Reactive Protein assay (Reagent OSR6199) on the AU480 Chemistry Analyzer (Beckman Coulter, Brea, CA, USA). Hereafter, we refer to these measurements as first-trimester CRP. As the CRP levels exhibited a left-skewed distribution, a log transformation was applied to normalize the values for subsequent analysis.

Genome-wide genotyping was conducted on 9,757 mothers with whole blood sample using the Infinium Multi-Ethnic Global D2 BeadChip (Illumina, Miami, FL, USA) [25]. Quality control (QC) and genotype imputation were applied, following the pipeline detailed in Yan et al. (2023) [26]. QC procedures included checking for genotyping call rates, Hardy-Weinberg equilibrium, and minor allele frequency. A total of 9,742 women had genotying data that passed QC [26], and 6,669,023 common variants (minor allele frequency (MAF) * >0.05*) were available for analysis. The final dataset used for analysis included 4,326 mothers with both genetic data and CRP measurements available. Genome-wide genotyping was performed on 3,860 offspring using DNA extracted from cord blood collected at birth, with the Illumina Multi-Ethnic Global Array (v8) [27]. After genotype imputation using the TOPMed imputation server with the TOPMed r3 reference panel [28], we applied QC procedures, including imputation quality score (r^2^) > 0.7, MAF > 5%, a Hardy–Weinberg Equilibrium (HWE) P-value > 1 × 10⁻⁶, genotyping call rate > 99% per individual and per marker, and concordance between reported and genetic sex. 2,140 offspring had matched maternal CRP data, with 5,791,060 common variants available for analysis. All genotype positions were based on the GRCh38 (hg38) genome build.

GDM cases were identified based on clinical glucose testing or a documented clinical diagnosis. Participants met GDM criteria if they had: (1) two abnormal values on a 100 g 3-hour OGTT, (2) one abnormal value on a 75 g 2-hour OGTT, (3) a 50 g glucose challenge result ≥ 200 mg/dL without follow-up testing, or (4) a GDM diagnosis recorded in the medical chart; individuals with pregestational diabetes were excluded, and the remaining participants were classified as controls.

### Statistical analysis

The ancestry of the 4,326 multi-ancestry maternal participants was determined using SNPweights v2.1 [29], based on reference data from the 1000 genome project consortium [30]. We assigned ancestry if the predicted ancestry proportion exceeded 80% [25], resulting in classification into six ancestral groups: African (AFR, *n* = 349), Admixed American (AMR, *n* = 162), East Asian (EAS, *n* = 84), European (EUR *n* = 2,749), South Asian (SAS, *n* = 22), and Unassigned Ancestry (*n* = 960). We primarily investigated the genetic contributions of first-trimester CRP in European-ancestry cohort, given its larger sample size and population homogeneity. For this analysis, we restricted to unrelated individuals of European ancestry (*n* = 2,736) to avoid confounding due to relatedness, and performed GWAS using PLINK v2.0 [31]. For the multi-ancestry cohort, GWAS was performed using the GENESIS [32], which accounts for population structure and relatedness using a linear mixed-effects model. In both analyses, covariates included standardized age, standardized age squared, 10 principal components (PCs) for genetic ancestry, and clinical sites.

Fine-mapping was performed using SuSiE-inf [33] with in-sample LD calculated using PLINK v1.9 [34]. Fine-mapping regions were defined as ±1 MB around each lead variant, allowing for up to five independent signals per region. For each signal, SuSiE-inf identified a 95% credible set containing variants that together account for 95% of the posterior probability of causality. For functional downstream analysis, we used Functional Mapping and Annotation (FUMA) tool v1.6.3 [35], which requires input data aligned to the GRCh37 genome build. Therefore, the GWAS summary statistics based on GRCh38 were lifted over to GRCh37 using the UCSC liftOver tool [36] to ensure compatibility.

To estimate the genome-wide genetic correlation between CRP and GDM, we applied LDSC [37], which assesses the extent of shared genetic architecture between two traits using GWAS summary statistics. The primary analysis used summary statistics from the nuMoM2b CRP GWAS and a large GDM GWAS (*n* = 143,441) in Elliott et al. [38]. As a complementary analysis, we repeated LDSC using CRP summary statistics from a large population-based GWAS by Said et al. [16], paired with the same GDM dataset.

Additionally, to evaluate whether genetic predisposition to elevated CRP levels contributes to GDM risk, we constructed PRS using the clumping and thresholding approach [39] in PLINK v1.9 [34]. SNP weights were derived from the same two CRP GWAS datasets described above. LD clumping was performed using the nuMoM2b genotype dataset as the LD reference panel with an r^2^ threshold of 0.1 within a 250-kb window. Because of the limited sample size of the nuMoM2b GWAS, a more lenient suggestive significance threshold of P < $\:1\times\:{10}^{-5}$ was used to to retain a sufficient number of variants for PRS construction and improve stability. For the external CRP GWAS, genome-wide significant variants (P < $\:5\times\:{10}^{-8}$) were used. Associations between PRS and GDM status were evaluated using unadjusted logistic regression, focusing on the marginal effect of PRS on GDM risks.

To evaluate potential causal effects of CRP on GDM risk, we conducted two-sample MR analyses using CRP summary statistics from Said et al. [16] as the exposure dataset and the GDM GWAS from Elliott et al. [38] as the outcome dataset. CRP-associated variants reaching genome-wide significance (*P* < 5 × 10⁻⁸) were selected as instrumental variables and pruned for linkage disequilibrium using LD clumping. To reduce potential pleiotropic effects, we performed a cis-MR analysis restricting variants to those located within ± 1 Mb of the CRP gene locus. The inverse-variance weighted (IVW) method was used as the primary estimator, with MR-Egger, weighted median, weighted mode, simple mode, and MR-RAPS as sensitivity analyses. Horizontal pleiotropy and heterogeneity were assessed using the MR-Egger intercept and Cochran’s Q statistic.

**Fig. 1:**
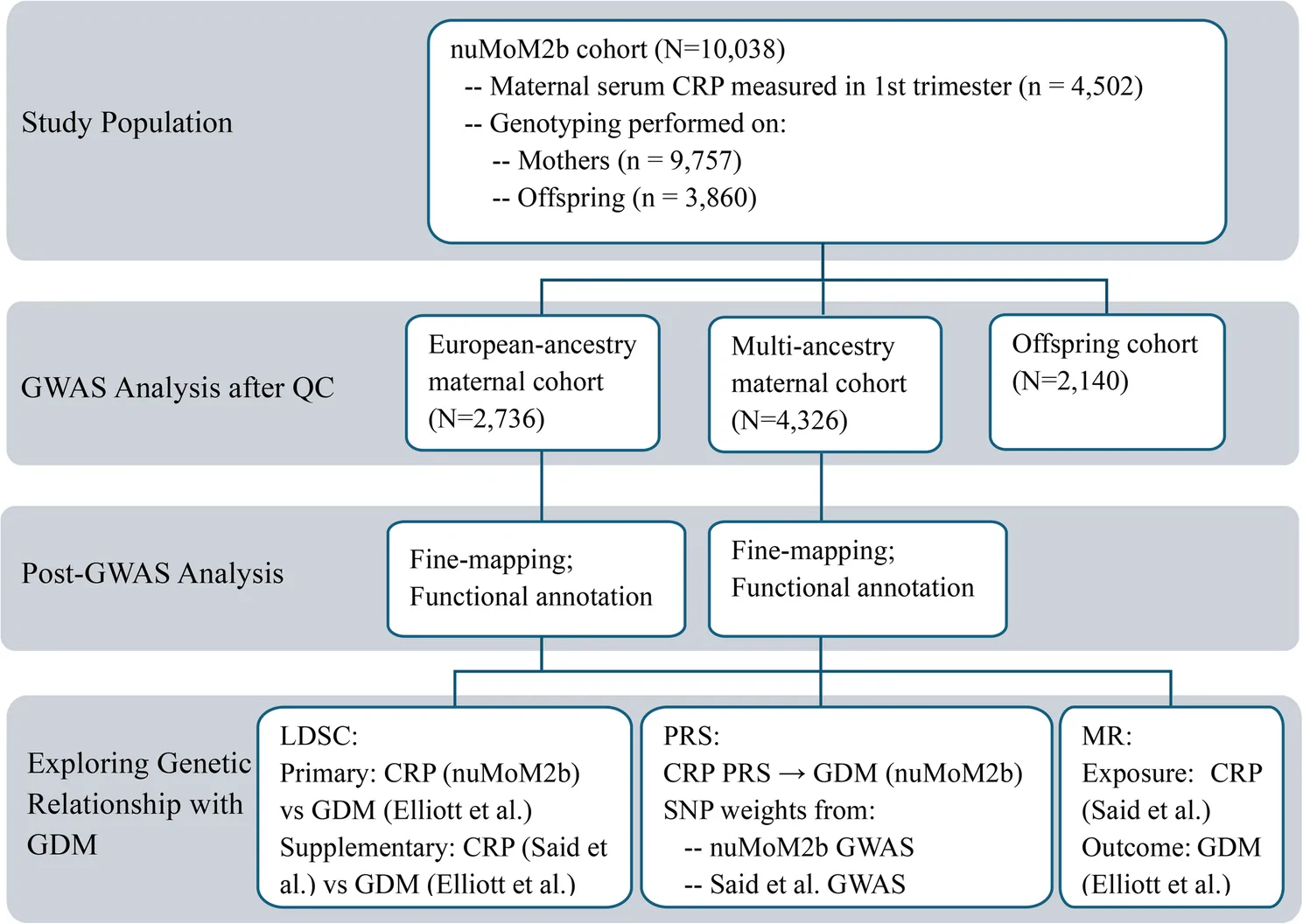
Overview of study design and analysis workflow. GDM: Gestational diabetes mellitus; LDSC: LD Score Regression; PRS: Polygenic risk score

Finally, to investigate the potential contribution of offspring genetic variation to maternal CRP levels, we performed an offspring-based GWAS of maternal first-trimester CRP. Due to the limited sample size of available offspring genotype data, this analysis was conducted using the full multi-ancestry sample in GENESIS, adjusting for the same covariates as in the maternal analysis, with the addition of offspring sex. The genome-wide significance (GWS) level was set at 5 × 10^− 8^. The overall study and analysis workflow is illustrated in Fig. 1.

## Results

### European-ancestry maternal cohort

#### GWAS of first-trimester CRP levels

**Fig. 2:**
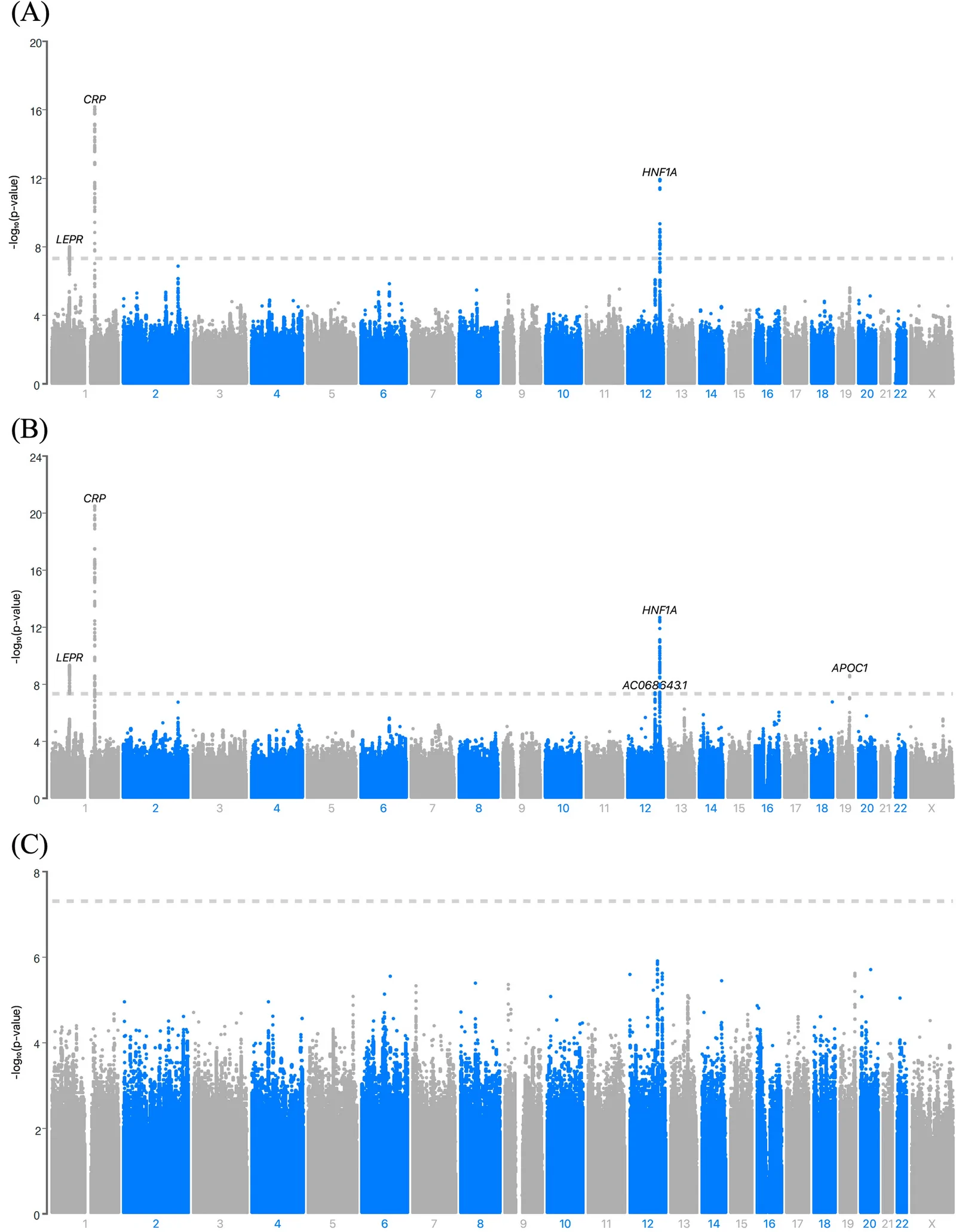
Manhattan plot of GWAS of first-trimester CRP. -log10 P values on the y axis are plotted against physical position (GRCh38) on the x axis. The horizontal dashed line represents the significance level (*P* < 5 × 10^− 8^). **A** European-ancestry maternal cohort. **B** multi-ancestry maternal cohort. **C** multi-ancestry offspring cohort

**Fig. 3:**
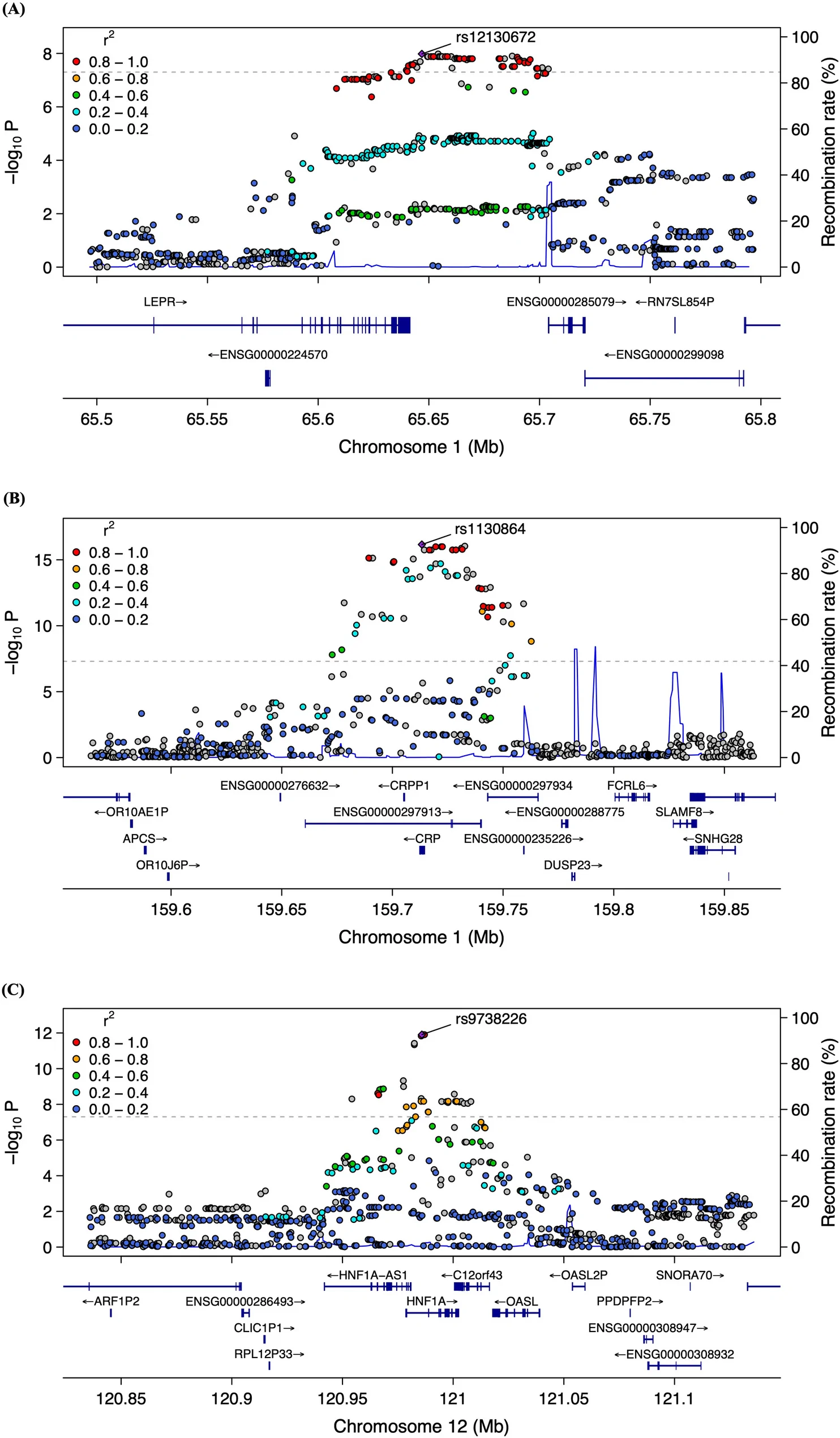
Regional plots of genome-wide significant loci in the European-ancestry maternal cohort. **A** Regional plot for SNP rs12130672. **B** Regional plot for SNP rs1130864. **C** Regional plot for SNP rs9738226. The lower panel of each plot shows the relative location of nearby genes and their transcriptional direction. The lead SNP is highlighted as a purple diamond, while surrounding SNPs are color-coded based on their Linkage Disequilibrium (LD, r²) with the lead SNP, calculated using the 1000 Genomes European reference pane

Three genome-wide significance (GWS) loci including 166 genetic variants were identified in the European-ancestry maternal cohort within the nuMoM2b study (Fig. 2A, Additional File 1: Table S1). The genomic inflation factor (λ = 1.01) suggests minimal inflation of test statistics due to population stratification or other confounding factors, as further supported by the QQ plot (Additional File 2: Fig S1). Additionally, principal component analysis (PCA) confirmed genetic ancestry consistency within the cohort (Additional File 1: Fig S2). Among the 166 genome-wide significant variants, the strongest association signal was rs1130864, an intronic variant within the *CRP* gene on chromosome 1 (β = 0.25, *P* = 6.94 × 10^–17^; Fig. 3B). A second locus on Chromosome 1 was marked by lead SNP rs12130672, located near *LEPR* (β = -0.16, *P* = 1.31 × 10^− 8^; Fig. 3A). The third locus, on chromosome 12, spans the *HNF1A* and *C12orf43* genes and is marked by lead SNP rs9738226, which lies within *HNF1A* (β = -0.20, *P* = 1.24 × 10^–12^; Fig. 3C). Fine-mapping analysis identified four 95% credible sets (Additional File 1: Table S2). Three of these overlapped with the genome-wide significant loci identified in the GWAS. One credible set, however, included SNPs that did not reach genome-wide significance but were prioritized based on high posterior probabilities. These SNPs are located within *ENSG00000297913*, a long non-coding RNA (lncRNA) positioned antisense to the *CRP* gene.

#### Functional downstream analysis

Functional annotation of GWS variants and their linkage disequilibrium (LD)-linked variants (Additional File 1: Table S3) revealed two variants on chromosome 1: rs876537 (ncRNA_exonic *CRPP1*) and rs12083620 (intergenic), both with combined annotation-dependent depletion (CADD) scores above 12.37. Regulatory annotation using RegulomeDB score identified rs4656849 (intergenic), rs7953249 (intergenic) and rs1169310 (UTR3 *HNF1A*) as variants with regulatory potential. Furthermore, Functional Mapping and Annotation (FUMA) [35] ANNOVAR analysis showed that over 80% of GWS SNPs and their LD-linked variants were in intergenic and intronic regions (Additional File 2: Fig S3). Those GWS variants were also mapped to 98 protein-coding genes (Additional File 2: Table S4) via the positional mapping, expression quantitative trait loci (eQTL), and chromatin interaction mapping. Not only the proximal genes but also the distal genes were identified with eQTL/chromatin interaction mapping (Additional File 2: Figs S4 and S5). All the variants in GWAS were annotated to 19,273 protein coding genes using FUMA-MAGMA, of which three genes (*CRP*, *HNF1A*, *C12orf43*) are associated with first-trimester CRP at Bonferroni significance level (*P* < 2.60 × 10^− 6^) (Additional File 2: Fig S6, Additional File 1: Table S5). FUMA-MAGMA tissue expression analysis identified enrichment of first-trimester CRP associated genes in kidney, fallopian tube, pancreas, and heart tissues (Additional File 2: Fig S7), although they did not reach Bonferroni-corrected statistical significance (*P* ≤ 1.67 × 10^− 3^).

#### Genetic relationship between CRP and GDM

SNP-based heritability of first-trimester CRP estimated from the European-ancestry GWAS was estimated at 30.3% (SE = 16.4%). Using LDSC, we assessed the genome-wide genetic correlation between CRP and GDM based on our first-trimester CRP GWAS in nuMoM2b and the external GDM GWAS from Elliott et al. [38]. This pregnancy-specific analysis did not detect a significant genetic correlation (r_g_ = 0.044, SE = 0.221, *P* = 0.841). In contrast, when CRP summary statistics from a larger population-based GWAS [16] were used, a modest genetic correlation with GDM was observed (r_g_ = 0.197, SE = 0.058, *P* = 0.0007).

we next evaluated whether the genetic predisposition to elevated CRP levels was associated with GDM risk in the nuMoM2b cohort using PRS derived from both CRP GWAS sources, respectively. The PRS constructed from the nuMoM2b CRP GWAS included 20 variants and was not significantly associated with GDM (β = 0.165, *P* = 0.405), explaining a negligible proportion of variance in GDM risk (Nagelkerke $\:{R}^{2}$= 0.00091). Similarly, the PRS derived from a large external CRP GWAS included 768 variants and was also not significantly associated with GDM (β = −0.158, *P* = 0.247), with minimal variance explained (Nagelkerke $\:{R}^{2}$ = 0.00175). Because the PRS derived from the nuMoM2b CRP GWAS was constructed and evaluated within the same cohort, these results should be interpreted cautiously due to potential overfitting.

We further investigated the potential causal effect of CRP on GDM risk using cis-MR. Eight independent CRP-associated variants located within ± 1 Mb of the CRP gene locus were used as instrumental variables. The primary inverse-variance weighted (IVW) analysis did not support a causal effect of genetically predicted CRP on GDM risk (β = 0.093, SE = 0.174, *P* = 0.594). Consistent null results were observed across sensitivity analyses, including weighted median, MR-Egger, weighted mode, simple mode, and MR-RAPS (all *P* > 0.05). Diagnostic tests indicated no evidence of directional pleiotropy (MR-Egger intercept *P* = 0.722) or significant heterogeneity among instrumental variables (Cochran’s Q *P* = 0.245).

### Multi-ancestry maternal cohorts

#### GWAS of first-trimester CRP levels

**Fig. 4:**
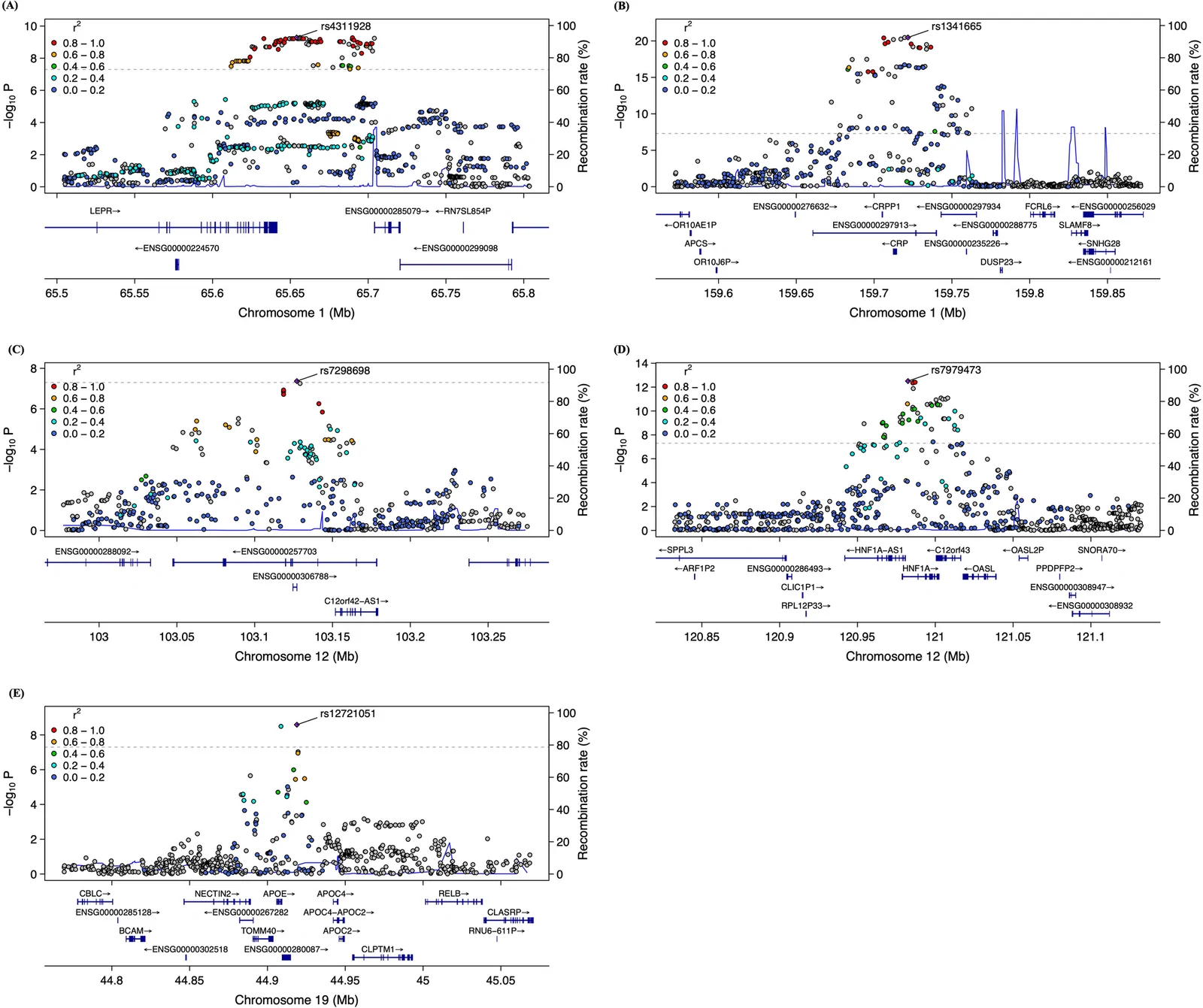
Regional plot for genome-wide significant loci in the multi-ancestry maternal cohort. **A** Regional plot for SNP rs4311928. **B** Regional plot for SNP rs1341665. **C** Regional plot for SNP rs7298698. **D** Regional plot for SNP rs7979473. **E** Regional plot for SNP rs12721051. The lower panel of each plot shows the relative location of nearby genes and their transcriptional direction. The lead SNP is highlighted as a purple diamond, while surrounding SNPs are color-coded based on their Linkage Disequilibrium (LD, r²) with the lead SNP, calculated using the 1000 Genomes reference panel (ALL populations)

In the multi-ancestry maternal cohort, five GWS loci including 237 genetic variants were identified in multi-ancestry cohort (Fig. 2B, Additional File 1: Table S6). The genomic inflation factor (λ = 1.01) and corresponding QQ plot (Additional File 1: Fig S8) suggest minimal inflation of association statistics, indicating limited population stratification or other confounding influences. The strongest association was observed for rs1341665, an intergenic variant on chromosome 1q23.2 near the *CRP* gene (β = -0.23, *P* = 3.36 × 10^–21^; Fig. 4B). On the same chromosome, the top variant rs4311928 near *LEPR* also reached genome-wide significance (β = -0.14, *P* = 5.12 × 10^–10^; Fig. 4A). Two additional loci were identified on chromosome 12, marked by lead variant rs7298698 within *ENSG00000257703* (also known as *AC068643.1*) (β = 0.12, *P* = 4.27 × 10^− 8^; Fig. 4C) and rs7979473, an intronic variant in *HNF1A* (β = -0.17, *P* = 2.21 × 10^–13^; Fig. 4D). The final significant locus was located on chromosome 19, marked by rs12721051, an intronic variant in *APOC1* (β = -0.19, *P* = 2.59 × 10^− 9^; Fig. 4E). Fine-mapping analysis identified six 95% credible sets across the five GWS loci (Additional File 1: Table S7). One of the credible sets consisted of a single variant (rs2369146) that did not overlap with a GWS locus but was highly prioritized based on posterior probability.

#### Functional downstream analysis

Applying the same annotation framework to multi-ancestry GWAS, we identified 11 prioritized variants with CADD scores above 12.37 (Additional File 1: Table S8). RegulomeDB scores annotated six variants with potential regulatory roles, including four intergenic variants (rs4656849, rs6489786, rs7953249, rs2251468), one variant in the 3’ UTR of *HNF1A* (rs1169310) and one intronic variant in *C12orf43* (rs1169314). Consistent with the European-ancestry results, the majority of GWS SNPs and their LD-linked variants were located in intergenic and intronic regions (Additional File 2: Fig S9). Those GWS variants were mapped to 119 protein-coding genes (Additional File 1: Table S9) via the positional mapping, expression quantitative trait loci (eQTL) and chromatin interaction mapping, capturing both nearby and distal gene relationships (Additional File 2: Figs S10-S12). Gene-level analysis using FUMA-MAGMA identified five genes (*CRP*, *HNF1A*, *C12orf43*, *APOE*, *APOC1*) associated with first-trimester CRP at Bonferroni significance (*P* < 2.58 × 10^− 6^) (Additional File 2: Fig S13, Additional File 1: Table S10). Tissue enrichment analysis suggested enrichment in liver tissues (Additional File 2: Fig S14), although they did not reach Bonferroni-corrected significance (*P* < 1.67 × 10^− 3^).

#### Genetic relationship between CRP and GDM

SNP-based heritability of first-trimester CRP estimated from the multi-ancestry GWAS was 16.3% (SE = 9.2%). Applying the same LDSC framework, we evaluated the genome-wide genetic correlation between CRP and GDM using the multi-ancestry CRP GWAS from the nuMoM2b cohort and the external GDM GWAS from Elliott et al. [38]. Consistent with the European-ancestry analysis, no significant genetic correlation was observed (r_g_ = 0.264, SE = 0.206, *P* = 0.200).

Using a similar analytical approach, we evaluated the association between genetically predicted CRP levels and GDM risk in the nuMoM2b multi-ancestry cohort using PRS derived from both CRP GWAS sources. The PRS based on the multi-ancestry nuMoM2b CRP GWAS (24 variants) was not significantly associated with GDM (β = 0.055, *P* = 0.756) and explained a negligible proportion of variance (Nagelkerke R² = 0.000091). Consistently, the PRS derived from a large external CRP GWAS (857 variants) also showed no significant association (β = −0.171, *P* = 0.113) and minimal variance explained (Nagelkerke R² = 0.0024). As the PRS derived from the nuMoM2b multi-ancestry GWAS was constructed and evaluated within the same cohort, these results should be interpreted with caution due to potential overfitting.

### Offspring cohorts

In the offspring GWAS of maternal CRP levels, no variants reached genome-wide significance (*P* < 5 × 10^− 8^, Fig. 2C). However, 42 variants demonstrated suggestive associations at a less stringent threshold (*P* < 1 × 10^− 5^, Additional File 1: Table S11). Notably, some of these variants were located in or near genes previously implicated in inflammatory processes, such as *HNF1A* and *FBLN5* [40]. An interactive plot showing all association results is available at https://my.locuszoom.org/gwas/130031/?token=4c1783ffb536418da22d63abaeb7d305 for further exploration.

## Discussion

CRP is a circulating, nonspecific marker of systemic inflammation that is primarily synthesized by hepatocytes [3]. Although the liver is the predominant source, extrahepatic production of CRP has been reported, including endothelial cells, smooth muscle cells, and the placenta [41–43]. The expression of CRP is induced mainly by interleukin‑6 (IL-6) and is further amplified by IL-1β, making these cytokines the central regulators of the acute‑phase response. IL-6 acts as the primary inducer of CRP transcription by activating STAT3, which is a transcription factor that binds to regulatory elements in the proximal CRP promoter [44]. IL-1β, in turn, potentiates the effects of IL-6-mediated effects by increasing IL-6 receptor expression and enhancing signaling through C/EBP-β pathway [45, 46]. Pregnancy is associated with dynamic changes in circulating inflammatory signaling, along with physiologic changes in plasma volume and renal clearance [47]. CRP levels are higher in pregnant than in non‑pregnant individuals and increase with advancing gestational age [13, 48]. In a recent longitudinal cohort of 707 pregnant individuals, CRP increased steadily throughout gestation, whereas multiple cytokines followed distinct, and often markedly decreasing trajectories through pregnancy and surged only after pregnancies progressed beyond term, indicating that there is potentially a pregnancy‑specific decoupling between rising hepatic CRP output and the trajectories of individual circulating cytokines [49]. Further supporting the non‑parallel behavior of CRP and cytokines, longitudinal profiling in pregnancies with and without polycystic ovary syndrome (PCOS) showed distinct gestational trajectories of CRP and 22 cytokines, with early immune activation particularly pronounced in PCOS, underscoring that maternal metabolic/endocrine context can modulate these inflammatory patterns [50]. Together, these considerations underscore the need to delineate the genetic architecture of CRP specifically in pregnancy, motivating our GWAS of first‑trimester CRP to identify loci that regulate maternal inflammation in this unique physiological state.

In this comprehensive GWAS investigating the genetic contributions to CRP levels during early pregnancy, we analyzed both maternal and offspring genotypes from the nuMoM2b cohort. Consistent with previous large-scale CRP GWAS conducted in general European-ancestry populations [16], we identified three GWS loci in the European-ancestry maternal cohort, including variants within or near the *CRP*, *LEPR*, *HNF1A* and *C12orf43* genes. These loci are well-established regulators of systemic inflammation and metabolic homeostasis [51, 52]. For example, *LEPR*, the leptin receptor, is ubiquitously expressed receptor involved in regulating energy balance, appetite, and immune function [53, 54]. Polymorphisms or mutations in the *LEPR* gene are associated with changes in diet and obesity [55–57]. *HNF1A*, the (hepatocyte nuclear factor 1 homeobox A), is a transcription factor that is expressed in many tissues including pancreas, liver, and kidney [58]. Mutations in *HNF1A* is also known to cause a monogenic form of diabetes and polymorphisms have been linked to risk of GDM [59–62]. While the number of genome-wide significant variants was much smaller than those reported in the large general European study, the overall consistency in locus-level associations reinforces the robustness of our findings. Fine-mapping analyses further identified additional candidate variants, including those in non-coding regions such as *ENSG00000297913*, a lncRNA transcribed antisense to the *CRP* gene. Antisense lncRNAs can regulate gene expression through transcriptional interference, RNA stability, or chromatin remodeling mechanisms [63], suggesting that these variants may modulate CRP levels even in the absence of genome-wide significance.

Notably, two additional GWS loci—marked by lead SNPs rs7298698 (within *ENSG00000257703*) and rs12721051 (within *APOC1*)—were identified in the multi-ancestry cohort beyond those detected in the European-ancestry analysis. Although both variants had similar effect sizes and allele frequencies across the two cohorts (e.g., rs7298698: β = 0.124, MAF = 0.48 in multi-ancestry vs. β = 0.137, MAF = 0.54 in European-ancestry; rs12721051: β = -0.189, MAF = 0.144 in multi-ancestry vs. β = -0.171, MAF = 0.173 in European-ancestry), they reached genome-wide significance only in the larger, multi-ancestry sample, likely due to increased statistical power. This highlights the importance of diverse cohorts in uncovering associations that may be missed in ancestry-restricted analyses. Additional ancestry-specific analyses further evaluating these loci are provided in Additional file 2. Functionally, this region on chromosome 19 includes both *APOC1* and *APOE*, which are closely linked and have been implicated in lipid metabolism, inflammation, and cardiovascular risk [64]. In contrast, *ENSG00000257703* encodes a long non-coding RNA with unclear biological function but consistent association signals in CRP GWAS [16], suggesting a possible regulatory role that needs further investigation.

Although no loci reached genome-wide significance in the offspring GWAS of maternal CRP during pregnancy, several loci exhibited suggestive associations (*P* < 1 × 10^− 5^). Given the relatively small offspring sample size (*n* = 2140), these findings should be interpreted as exploratory. Prior studies have reported offspring genetic effects on maternal phenotypes, including blood pressure and glucose metabolism [19–21]. Such effects are thought to arise from placental signaling or fetal–maternal immunologic interactions during gestation. However, our study was underpowered to detect genome-wide significant associations, and the biological relevance of the suggestive loci remains uncertain. Larger studies with more extensive offspring genotype data will be necessary to clarify whether offspring genetic variation contributes to maternal CRP levels during pregnancy.

Finally, genetic correlation analysis using our first-trimester CRP GWAS in nuMoM2b did not detect significant evidence of shared heritability with GDM. In contrast, a complementary analysis using a much larger, population‐based CRP GWAS found a modest correlation. The modest genetic correlation observed in the general population may indicate some degree of shared genetic architecture between CRP and GDM; however, this finding may not directly translate to pregnancy-specific physiology. The absence of significant correlation in nuMoM2b may be attributable to limited statistical power given the smaller sample size. Additionally, our analysis was restricted to common variants (MAF > 0.05) and therefore may not capture potential contributions from rare variants. Furthermore, the PRS for CRP was not statistically associated with GDM in nuMoM2b. Consistent with this finding, MR analysis using variants within the CRP gene locus did not provide evidence supporting a causal effect of genetically predicted CRP on GDM risk. However, given the limited sample size of nuMoM2b used for PRS and number of cis-acting variants available as instruments, both approaches may be underpowered to detect modest genetic or causal effects, and the null findings should therefore be interpreted with caution.

Taken together, these genetic analyses do not provide strong support for a direct role of genetically determined CRP levels in the development of GDM within this cohort. However, these null results should be interpreted cautiously, as modest shared genetic effects may remain undetected due to limited sample size. The observed phenotypic association between early pregnancy CRP and GDM may reflect pregnancy-related inflammatory or metabolic changes, environmental influences such as obesity, diet, or stress, or complex interactions between genetic and non-genetic factors. While CRP may serve as a clinical indicator of underlying metabolic or inflammatory states, additional studies with larger and more diverse pregnancy cohorts will be necessary to clarify its potential mechanistic role in GDM pathogenesis.

While the modest sample size of nuMoM2b limits power to detect variants with smaller or rare effects on first-trimester CRP levels, the study’s strength lies in its uniquely rich design. nuMoM2b is one of the largest U.S. prospective pregnancy cohorts with biospecimens collected in the first trimester and includes both maternal and offspring genotypes across multiple ancestries. This enables the investigation of pregnancy-specific genetic architecture of CRP—a rare opportunity not captured in large general population studies. These features make the dataset uniquely valuable for advancing our understanding of maternal inflammation during early pregnancy. Consistent with this, independent pregnancy cohorts with comparable early gestation biomarker measurements and genome-wide genotype data are currently limited, which constrained our ability to perform external replication. Future studies in independent pregnancy cohorts will be important to validate these findings.

## Conclusions

By validating known systemic-inflammation loci (e.g., *CRP*, *LEPR*, *HNF1A*, *APOC1*) in pregnant women, exploring suggestive signals in offspring, we deliver the first gestation‐tailored genetic map of CRP regulation. These results not only fill a critical gap in the literature but also supply pregnancy‐specific data for future meta‐analyses and PRS construction, laying essential groundwork for subsequent studies with larger and more diverse cohorts.

## Supplementary Information

Supplementary Material 1.

Supplementary Material 2.

## Data Availability

Access to the nuMoM2b individual-level data is available through dbGaP under study accession phs002808.v1.p1 and the NICHD repository DASH. Interactive visualizations of the GWAS results can be accessed at: European-ancestry maternal GWAS [https://my.locuszoom.org/gwas/612155/?token=cd3ac564de804ec9afb1ee733e345f4c]; Multi-ancestry maternal GWAS [https://my.locuszoom.org/gwas/833469/?token=017c786d7c8640c8913c5c87b75378c9]; Offspring GWAS [https://my.locuszoom.org/gwas/130031/?token=4c1783ffb536418da22d63abaeb7d305].
