# Supplementary material for: Maternal and offspring genome-wide association study of C-reactive protein reveals limited polygenic association with gestational diabetes mellitus
Source: BMC Genomics. 2026 Sep 24;27:782. doi: 10.1186/s12864-026-12878-6 (PMC13602706; doi:10.1186/s12864-026-12878-6)
Supplement: Supplementary file 1 [file 12864_2026_12878_MOESM2_ESM.docx]

Supplements

# Results for European maternal cohort

Fig S1. QQ plot of European cohort (λ = 1.01).

Fig S2. The first two principal components (PC) from PCA using the 1000G against the nuMoM2b European cohort. The colored symbols represent the first two PCs of the individuals from different ancestries (EUR=European, EAS=East Asian, AMR=Ad Mixed American, AFR=African, SAS=South Asian) in the 1000G data, while the black crosses represent the first two PCs of the individuals in the nuMoM2b European ancestry cohort (OWN=nuMoM2b).

Fig S3. Functional consequences of SNPs on genes. The histogram displays the proportion of SNPs which have corresponding functional annotation assigned by ANNOVAR. Bars are colored by log2(enrichment) relative to all SNPs in the selected reference panel.

Fig S4. Circos plots of chromatin interactions and eQTLs on chromosome 1. The outermost layer is a Manhattan plot (GRCh37), where only SNPs with P < 0.05 are displayed. SNPs in genomic risk loci are color-coded as a function of their maximum r2 to the one of the independent significant SNPs in the locus, as follows: red (r2 > 0.8), orange (r2 > 0.6), green (r2 > 0.4) and blue (r2 > 0.2). SNPs that are not in LD with any of the independent significant SNPs (with r2 ≤ 0.2) are grey. Genomic risk loci are highlighted in blue in the second layer. Only mapped genes by either chromatin interaction and/or eQTLs are displayed. If the gene is mapped only by chromatin interactions or only by eQTLs, it is colored orange or green, respectively. When the gene is mapped by both, it is colored red. Chromatin interactions links are colored orange while eQTLs interaction links are colored green.

Fig S5. Circos plots of chromatin interactions and eQTLs on chromosome 12. The outermost layer is a Manhattan plot (GRCh37), where only SNPs with P < 0.05 are displayed. SNPs in genomic risk loci are color-coded as a function of their maximum r2 to the one of the independent significant SNPs in the locus, as follows: red (r2 > 0.8), orange (r2 > 0.6), green (r2 > 0.4) and blue (r2 > 0.2). SNPs that are not in LD with any of the independent significant SNPs (with r2 ≤ 0.2) are grey. Genomic risk loci are highlighted in blue in the second layer. Only mapped genes by either chromatin interaction and/or eQTLs are displayed. If the gene is mapped only by chromatin interactions or only by eQTLs, it is colored orange or green, respectively. When the gene is mapped by both, it is colored red. Chromatin interactions links are colored orange while eQTLs interaction links are colored green.

Fig S6. Manhattan plot of the gene-based test as computed by FUMA-MAGMA based on the GWAS summary statistics.

Fig S7. FUMA-MAGMA Tissue Expression Analysis in GTEx v7 30 general tissue types in European maternal cohort.

# Results from multi-ancestry maternal cohort

Fig S8. QQ plot of multi-ancestry cohort (λ = 1.01).

Fig S9. Functional consequences of SNPs on genes. The histogram displays the proportion of SNPs which have corresponding functional annotation assigned by ANNOVAR. Bars are colored by log2(enrichment) relative to all SNPs in the selected reference panel.

Fig S10. Circos plots of chromatin interactions and eQTLs on chromosome 1. The outermost layer is a Manhattan plot (GRCh37), where only SNPs with P < 0.05 are displayed. SNPs in genomic risk loci are color-coded as a function of their maximum r2 to the one of the independent significant SNPs in the locus, as follows: red (r2 > 0.8), orange (r2 > 0.6), green (r2 > 0.4) and blue (r2 > 0.2). SNPs that are not in LD with any of the independent significant SNPs (with r2 ≤ 0.2) are grey. Genomic risk loci are highlighted in blue in the second layer. Only mapped genes by either chromatin interaction and/or eQTLs are displayed. If the gene is mapped only by chromatin interactions or only by eQTLs, it is colored orange or green, respectively. When the gene is mapped by both, it is colored red. Chromatin interactions links are colored orange while eQTLs interaction links are colored green.

Fig S11. Circos plots of chromatin interactions and eQTLs on chromosome 12. The outermost layer is a Manhattan plot (GRCh37), where only SNPs with P < 0.05 are displayed. SNPs in genomic risk loci are color-coded as a function of their maximum r2 to the one of the independent significant SNPs in the locus, as follows: red (r2 > 0.8), orange (r2 > 0.6), green (r2 > 0.4) and blue (r2 > 0.2). SNPs that are not in LD with any of the independent significant SNPs (with r2 ≤ 0.2) are grey. Genomic risk loci are highlighted in blue in the second layer. Only mapped genes by either chromatin interaction and/or eQTLs are displayed. If the gene is mapped only by chromatin interactions or only by eQTLs, it is colored orange or green, respectively. When the gene is mapped by both, it is colored red. Chromatin interactions links are colored orange while eQTLs interaction links are colored green.

Fig S12. Circos plots of chromatin interactions and eQTLs on chromosome 19. The outermost layer is a Manhattan plot (GRCh37), where only SNPs with P < 0.05 are displayed. SNPs in genomic risk loci are color-coded as a function of their maximum r2 to the one of the independent significant SNPs in the locus, as follows: red (r2 > 0.8), orange (r2 > 0.6), green (r2 > 0.4) and blue (r2 > 0.2). SNPs that are not in LD with any of the independent significant SNPs (with r2 ≤ 0.2) are grey. Genomic risk loci are highlighted in blue in the second layer. Only mapped genes by either chromatin interaction and/or eQTLs are displayed. If the gene is mapped only by chromatin interactions or only by eQTLs, it is colored orange or green, respectively. When the gene is mapped by both, it is colored red. Chromatin interactions links are colored orange while eQTLs interaction links are colored green.

Fig S13. Manhattan plot of the gene-based test as computed by MAGMA based on the GWAS multi-ancestry summary statistics.

Fig S14. MAGMA Tissue Expression Analysis in GTEx v7 30 general tissue types in multi-ancestry cohort.

# Ancestry-specific effect estimates for multi-ancestry GWAS loci

To further evaluate the two additional loci identified in the multi-ancestry GWAS analysis, we examined ancestry-specific effect estimates for the lead SNPs rs7298698 and rs12721051 across the major ancestry groups represented in the nuMoM2b cohort. Because the multi-ancestry analysis included individuals from several ancestral backgrounds, assessing whether the observed associations were consistent across ancestry groups can help clarify whether the signals reflect broadly shared genetic effects or ancestry-specific patterns.

We extracted ancestry-specific association results for European (EUR), African (AFR), and Admixed American (AMR) individuals using the same phenotype and covariate adjustments as in the primary analyses. Effect estimates were aligned to the same effect allele across ancestry groups (T for rs7298698 and G for rs12721051). The ancestry-specific association statistics, including sample size, minor allele frequency, effect size, standard error, and P value, are presented in Table S12. Forest plots summarizing the ancestry-specific effect estimates and corresponding 95% confidence intervals are shown in Fig S15. For comparison, the effect estimates from the overall multi-ancestry GWAS are also displayed in the figure.

To formally evaluate whether the effect sizes differed across ancestry groups, we conducted heterogeneity tests using Cochran’s Q statistic based on the ancestry-specific estimates (EUR, AFR, and AMR). No statistically significant heterogeneity was observed for either locus (rs7298698: Q = 3.69, df = 2, P = 0.158; rs12721051: Q = 0.15, df = 2, P = 0.926), suggesting no strong evidence of heterogeneity in effect estimates across ancestry group.

Because the non-European ancestry groups in the nuMoM2b cohort are relatively small, the ancestry-specific estimates have wider confidence intervals and should be interpreted cautiously. Nonetheless, the direction of effect was generally consistent across ancestry groups and aligned with the effect observed in the multi-ancestry analysis. These results suggest that the associations detected at rs7298698 and rs12721051 are unlikely to be driven by a single ancestry group within the cohort.

Fig S15. Forest plots of ancestry-specific effect estimates for the lead SNPs rs7298698 and rs12721051.
